# Annotations

**Published:** 1905-01-21

**Authors:** 


### ANNOTATIONS.

### Mr. Chamberlain and the Medical Profession.

DURING the period Mr. Chamberlain filled the post of Colonial Secretary he took frequent opportunities of manifesting his keen appreciation of the work of members of the medical profession in Greater Britain, and especially in the dependencies where pioneer efforts, under conditions of difficulty and danger, are carried on. At a meeting in connection with the Liverpool School of Tropical Medicine last week Mr. Chamberlain availed himself of the occasion to make it clear that his retirement from office has not in the least diminished his interest in the treatment of maladies peculiar to many of our colonial possessions. Imperialist as he himself is, Mr. Chamberlain realises the fact that we pay a heavy tribute for being an Imperial race, and that we lose, not only in military expeditions, but in the results of the endemic diseases which, as in vast areas of tropical Africa, paralyse administration and commerce, enormously increase the obstacles to the enterprise of the explorer and the mission of the religious teacher. For this reason, however, he hails as one of the founders of Empire the man who has the ability, the knowledge, and the courage to show how such losses can in any way be reduced. The compliment will not be the less acceptable because it is well merited. For the discoveries which have been made in respect to the nature of tropical diseases, of remedies which have not merely been the means of mitigating the sufferings of their victims, but of staying their ravages, though they may not be so showy as the feats of soldiers, or so sensational as the achievements of explorers, are even more calculated to be of world-wide benefit. Undertaken often enough in the face of constant privation and imminent peril, the expeditions on whose behalf Mr. Chamberlain claimed the admiration and the gratitude of the community, do not invariably obtain from statesmen and politicians the cordial recognition and the practical assistance which he has consistently accorded them.

### Bloodless Surgery.

THE inventor of this phrase, whoever he be, has hardly conferred a benefit on surgical practice or on that section of the public which is in need of surgical treatment. The specious and plausible qualities of the term made certain the ready welcome which it has received in popular regard. And the risks of popularity in medical names and methods have in this instance been fully realised. In its early day bloodless surgery caught the public ear in connection with the movements and practice of a surgeon who probably was far from desiring the notice he received in the lay press. But sensational journalism having once scented its prey never misses an opportunity to provide stimulation for the taste of its readers. More and more startling experiences are necessary to meet the increased craving for excitement. Hence from the comparative privacy of the hospital, bloodless surgery escaped into the open and was boomed with great success as the peculiar practice of a bone-setter in a Scottish mining village. Next appeared a rival practitioner of the art and lists of comparative claims and victories added the keen interest of competition and even of partisanship to the sensation. It is a matter for surprise that some or other "able editor" did not arrange for a challenge between the rivals and a test of skill to be conducted in his office with himself and his staff as arbiters. A further development was the appearance of bloodless surgery in Bond Street from which the clairvoyant and the magic crystal have so recently been evicted. And if the wall-posters are to be trusted it is shortly to make its appearance on the stage of the variety theatre. Such a record has many parallels and is not without its amusing side. But the chief thing to note is the perils which wait on a popularity based on ignorance and a love of sensation. No one familiar with the methods of the bone setter doubts that his rough proceedings sometimes get rid of adhesions and other conditions which mechanically interfere with free movement. But it is equally certain that he may and often does inflict serious damage. For this the sensation-monger must take his share of responsibility.

### The Habitual Drunkard.

THE habitual drunkard is a pathological as well as a social problem, and in the latest report of the inspector under the Inebriates Act there are several points of considerable interest both to the student of medicine and to the psychologist. First may be noted the rarity of serious organic disease amongst those formally and judicially certified as habitual inebriates. Though upwards of a thousand of the worst drunkards in the country have now been admitted to reformatories, there has not yet been observed among them a single typical instance of cirrhosis of the liver, and not one of them has required treatment for hæmatemesis, jaundice, ascites, or other evidence of serious portal obstruction. The explanation, it may be presumed, is that persons who do suffer these possible consequences of alcoholism die before they can be classed among the "habituals." It is a frequent experience to discover manifest evidences of cirrhosis of the liver and of portal obstruction in patients who, whilst admitting the free or even excessive use of alcoholism, deny intoxication at any time. The steady and systematic use of alcohol in relative excess, rather than occasional outbreaks of drunkenness with intervals of penitence and abstinence, is the condition which promotes the organic changes covered by the term alcoholism, and the habits of those who ultimately reach reformatory discipline are for the most part arranged less on the former than the latter plan. At the same time it must be recognised as surprising that, among a thousand persons described as "the worst drunkards in the country," there should be such scanty evidence of the pathological changes usually charged to the debit of alcohol. A second feature of importance in the inspector's report is found in the section dealing with the psychology of the certified inebriate. The conclusion presented is that drunkenness is to a large extent due to inherent mental defect, and that in very many cases the habitual drunkard only differs in degree from persons who can legally be termed of unsound mind. His mental disorder exhibits itself by insane actions and conduct rather than in the form of delusions and hallucinations.

